# Iatrogenic Kaposi’s sarcoma in myasthenia gravis: learnings from two case reports

**DOI:** 10.1007/s10072-020-04971-9

**Published:** 2021-01-06

**Authors:** Rita Frangiamore, Riccardo Giossi, Fiammetta Vanoli, Athanasia Tourlaki, Lucia Brambilla, Lorenzo Maggi, Renato Mantegazza

**Affiliations:** 1 Department of Neuroimmunology and Neuromuscular Diseases, Fondazione I.R.C.C.S. Istituto Neurologico Carlo Besta, Milano, Italy; 2 Department of Oncology and Onco-Hematology, Postgraduate School of Clinical Pharmacology and Toxicology, Università degli Studi di Milano, Milano, Italy; 3 Neuromuscular and Rare Disease Center, Department of Neuroscience, Mental Health and Sensory Organs (NESMOS), SAPIENZA University, Sant’Andrea Hospital, Rome, Italy; 4 U.O. Dermatologia, Fondazione IRCCS Ca’ Granda, Ospedale Maggiore Policlinico, Milano, Italy

**Keywords:** Myasthenia gravis, Kaposi’s sarcoma, Immunosuppressive agents, Corticosteroids, Case report

## Abstract

**Introduction:**

Myasthenia gravis (MG) is an autoimmune neuromuscular disease whose treatment encompasses acetylcholinesterase inhibitors, oral steroids, and other immunosuppressants. Kaposi’s sarcoma (KS) is a lymphangioproliferative disease associated with human herpesvirus 8 (HHV-8) infection and immunodeficiency or immunosuppression, mainly corticosteroids.

**Case reports:**

We present two cases of MG patients treated with oral steroids who developed KS. Patient 1 was diagnosed with three oral KS lesions. Prednisone was discontinued with lesion regression and stabilization, while azathioprine and pyridostigmine prompted control of MG. Patient 2 developed KS lesions on the trunk and lower limbs while taking prednisone and azathioprine. Steroid tapering was started but new oral and lymph nodal lesions appeared. Paclitaxel therapy was introduced and the patient experienced pulmonary embolism and developed sensitive neuropathy. Complete remission of KS lesions was achieved and maintained with azathioprine and pyridostigmine as MG medications.

**Conclusions:**

KS is an uncommon but clinically relevant adverse event (AE) often induced by steroid therapy. It can be controlled by steroid withdrawal but could necessitate chemotherapy, which associates with further potential AEs. Skin evaluation should be performed in all patients with chronic steroid therapy. Steroid-sparing strategies, including new drugs, could reduce KS and other steroid-related comorbidities. HHV-8 testing should be considered before starting chronic immunosuppression.

## Background

Myasthenia gravis (MG) is an autoimmune disease mostly associated with anti-acetylcholine receptor (AChR) antibodies. The clinical hallmark is weakness worsened by exercise. Therapy encompasses acetylcholinesterase inhibitors, oral steroids, and other immunosuppressants [1]. Kaposi’s sarcoma (KS) is a lymphangioproliferative disease associated with human herpesvirus 8 (HHV-8) infection and immunodeficiency or pharmacological immunosuppression, mainly corticosteroids [2]. Here, we describe two patients with MG who developed iatrogenic-KS (iKS). Both patients provided written informed consent.

## Case report

**Fig. 1:**
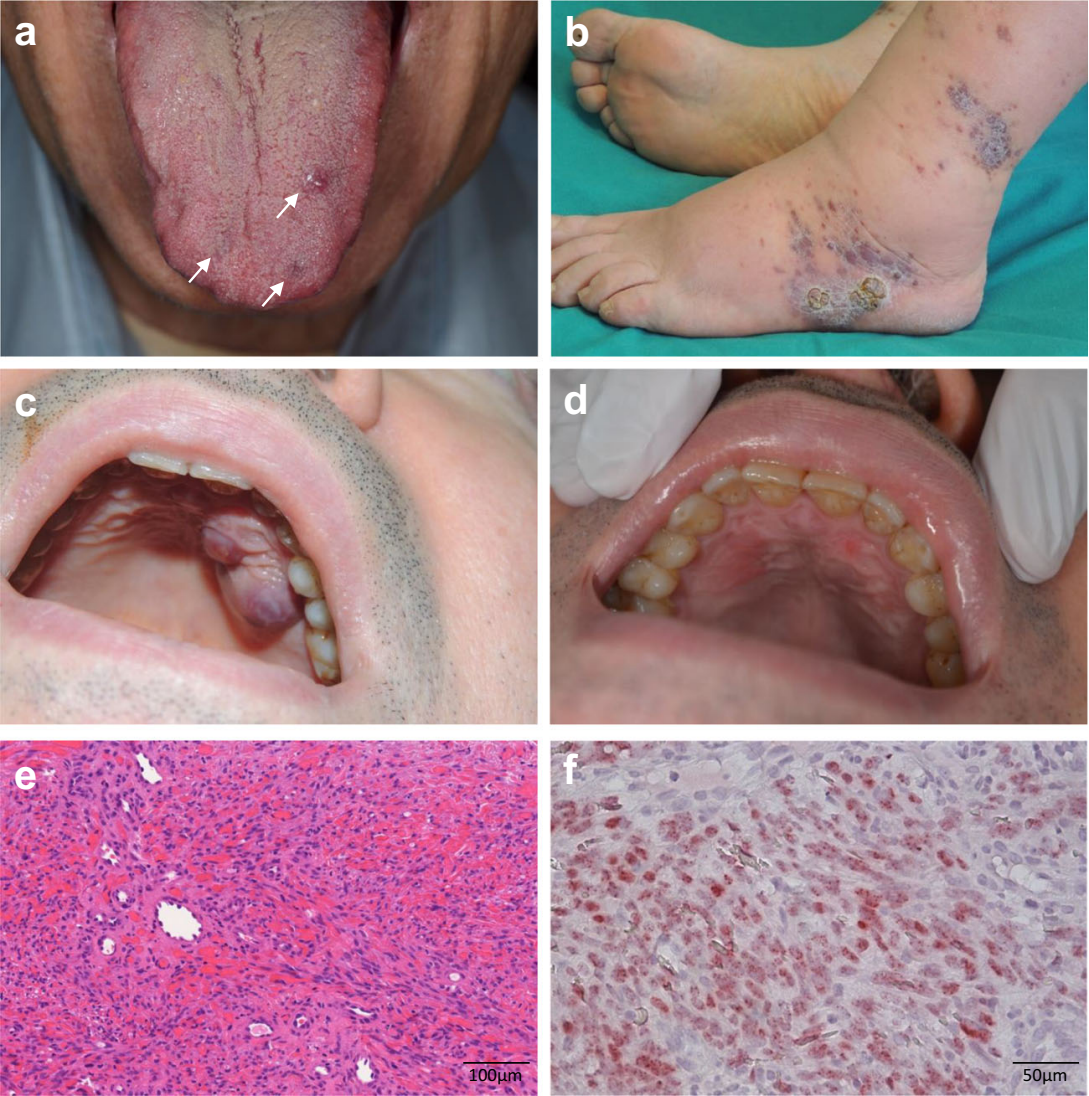
Clinical images showing Kaposi’s sarcoma lesions. **a** One nodule and two macules (arrows) on the tongue in Patient 1; **b** angiomatous nodules, plaques, and edema on the lower limbs in Patient 2; voluminous tumor on the hard palate in Patient 2 **c** before and **d** after steroid interruption and paclitaxel chemotherapy. **e** Hematoxylin-eosin stain of a biopsy specimen from Patient 2 showed proliferation of spindle cells and numerous extravasated erythrocytes between dermal collagen bundles. **f** The spindle cells stained positively with HHV-8 latency-associated nuclear antigen (LNA)-1

Patient 1 is a male diagnosed with anti-AChR-positive generalized MG (gMG) without thymus involvement, at 83 years of age (March 2015). As pyridostigmine provided partial improvement, prednisone 50 mg/day was initiated 6 months after diagnosis, with benefit. Body weight was 80 kg, corresponding to a 0.63 mg/kg/day dose of prednisone. Steroid-induced diabetes developed; hence, azathioprine was initiated, and prednisone tapered to 5 mg/day (May 2016), corresponding to a 0.06 mg/kg/day dose of prednisone. Body weight remained stable and steroid therapy maintained at this dosage. In September 2017, the patient was referred to a dermatologist to assess a small violaceous and two bluish macules on the tongue and hard palate (Fig. 1a). Biopsy analyses revealed typical KS features. Serology tested negative for HIV and positive for HHV-8. Staging examinations for KS, including fecal occult blood test, esophagogastroduodenoscopy, abdomen ultrasound, and otolaryngologic examination were negative [3]. iKS diagnosis was made and steroid was discontinued. Follow-up at 3, 6, and 12 months confirmed the presence of the small nodule on the tongue, while the two macules had completely regressed. To date, the nodule remains stable and MG is controlled with pyridostigmine and azathioprine 100 mg/day.

Patient 2 is a male diagnosed with anti-AChR-positive gMG at the age of 60 (October 2015), without thymus involvement. Pyridostigmine 60 mg four times daily (QID) and prednisone 65 mg/day were started. Body weight was 108 kg, corresponding to a 0.60 mg/kg/day dose of prednisone. In December 2015, azathioprine 150 mg/day was introduced and, due to limited efficacy, plasmapheresis was often used. Body weight was 100 kg, and prednisone 62.5 mg/day (0.63 mg/kg/day). In October 2016, the patient was referred to the dermatology department for numerous violaceous macules, plaques, and nodules on the lower limbs and reddish-purple plaques on the trunk. Bilateral ankle lymphoedema was present (Fig. 1b). A biopsy confirmed the diagnosis of KS, with serology positive for HHV-8 and negative for HIV. Staging examinations for KS were negative [3]. Whole blood count and biochemistry were normal. Patient body weight was 94 kg. MG therapy was prednisone 50 mg/day (0.53 mg/kg/day), azathioprine 150 mg/day, and pyridostigmine 90 mg QID. Upon iKS diagnosis, over the next 3 months, prednisone was tapered to 30 mg/day (0.32 mg/kg/day) and azathioprine was increased to 175 mg/day. Nevertheless, new KS lesions developed on the hard palate (Fig. 1c) and in left inguinal lymph nodes. Further lesions appeared on lower limbs and external ear during prednisone tapering to 5 mg/day. Thus, the patient stopped prednisone and started intravenous paclitaxel 100 mg/m^2^ weekly for 13 administrations with regression of all KS lesions (Fig. 1d). Five days after paclitaxel initiation, pulmonary embolism was detected and treated with enoxaparin with complete resolution. Moreover, the patient developed paclitaxel-induced axonal sensitive neuropathy. Three years after treatment, KS was still in remission, and MG symptoms were stable with azathioprine 200 mg/day plus pyridostigmine 60 mg QID.

## Discussion

Presented cases show the uncommon but clinically relevant adverse event of iKS, often induced by chronic steroid treatment. Notably, iKS generally occurs within the first 2 years of steroid treatment and about 8% of patients with iKS unrelated to organ transplants had MG [2]. iKS can be effectively controlled by steroid withdrawal (Patient 1). Additional chemotherapy is sometimes necessary, possibly causing additional severe comorbidities such as polyneuropathy and pulmonary embolism (Patient 2).

Oral corticosteroids are a cornerstone treatment in MG, though associated with relevant adverse events including iKS, interestingly even at low doses [2, 4, 5]. Notably, our patients received a maximum prednisone dose of 0.63 mg/kg/day. These doses are conformant to national recommendations of 0.75–1 mg/kg/day for initial gMG treatment, and even lower [6]. Patient 1 developed iKS when he was assuming 0.06 mg/kg/day of prednisone dose while being clinically stable for more than a year. Patient 2 developed iKS when he was slowly tapering prednisone (0.53 mg/kg/day) after the initial dose while being not clinically stable and often needing plasmaphereses.

iKS onset associates with immune-suppression through a direct effect on lymphangioendothelial cells, by downregulating the inhibition of endothelial cell growth [2]. It would be advisable to check for any suspicious skin or mucosal lesions, which are the most frequent localizations of iKS [2], in all patients on chronic corticosteroids. Moreover, considering the high seroprevalence of HHV-8 in regions such as the Mediterranean area (ranging 40 to 80%), it would be also advisable to test for HHV-8 to identify high-risk patients in which to possibly avoid corticosteroid treatment and to perform regular dermatologic assessments [7].

Reduction of iKS and other frequent steroid-related comorbidities might be achieved using new drugs, such as neonatal Fc receptor or complement inhibitors, which do not associate with reduced cellular immunity. As HHV-8 exploits the complement system to promote latent infection, targeting complement could be a useful therapeutic strategy but must be carefully investigated [8].

Awareness of iKS as a consequence of chronic corticosteroid treatment should be fostered and screening for HHV-8 considered before starting chronic immunosuppression.
